# Optimisation and evaluation of the random forest model in the efficacy prediction of chemoradiotherapy for advanced cervical cancer based on radiomics signature from high-resolution T2 weighted images

**DOI:** 10.1007/s00404-020-05908-5

**Published:** 2021-01-04

**Authors:** Defeng Liu, Xiaohang Zhang, Tao Zheng, Qinglei Shi, Yujie Cui, Yongji Wang, Lanxiang Liu

**Affiliations:** 1 Department of Magnetic Resonance Imaging, Qinhuangdao Municipal No. 1 Hospital, Qinhuangdao, People’s Republic of China; 2 State Grid Information & Telecommunication Group Co., Ltd., Beijing, People’s Republic of China; 3 Scientific Clinical Specialist, Siemens Ltd., Beijing, People’s Republic of China; 4 Cooperative Innovation Center, Institute of Software, Chinese Academy of Sciences, Beijing, People’s Republic of China; 5 University of Chinese Academy of Sciences, Beijing, People’s Republic of China; 6 State Key Laboratory of Computer Science (Institute of Software, The Chinese Academy of Sciences), Beijing, People’s Republic of China

**Keywords:** Random forest, Chemoradiotherapy, Radiomics, Cervical cancer, T2-weighted image

## Abstract

**Purpose:**

Our objective was to establish a random forest model and to evaluate its predictive capability of the treatment effect of neoadjuvant chemotherapy–radiation therapy.

**Methods:**

This retrospective study included 82 patients with locally advanced cervical cancer who underwent scanning from March 2013 to May 2018. The random forest model was established and optimised based on the open source toolkit scikit-learn. Byoptimising of the number of decision trees in the random forest, the criteria for selecting the final partition index and the minimum number of samples partitioned by each node, the performance of random forest in the prediction of the treatment effect of neoadjuvant chemotherapy–radiation therapy on advanced cervical cancer (> IIb) was evaluated.

**Results:**

The number of decision trees in the random forests influenced the model performance. When the number of decision trees was set to 10, 25, 40, 55, 70, 85 and 100, the performance of random forest model exhibited an increasing trend first and then a decreasing one. The criteria for the selection of final partition index showed significant effects on the generation of decision trees. The Gini index demonstrated a better effect compared with information gain index. The area under the receiver operating curve for Gini index attained a value of 0.917.

**Conclusion:**

The random forest model showed potential in predicting the treatment effect of neoadjuvant chemotherapy–radiation therapy based on high-resolution T2WIs for advanced cervical cancer (> IIb).

## Introduction

Cervical cancer, which is a main health problem for women, is one of the most common malignant tumours in gynaecology and ranks fourth among all malignant tumours [1–3]. According to the International Federation of Obstetrics and Gynaecology (FIGO) staging of cervical cancer, surgical resection is often used for early cervical cancer and concurrent chemoradiotherapy (CCRT) is often given clinically for middle and advanced cervical cancer, which usually loses the opportunity for radical surgical treatment. Although chemoradiotherapy improves the survival rate of advanced cervical cancer, several patients are still treated with poor efficacy [4]. If CCRT fails, the treatment options will be further limited and may result in increased toxicity and pathogenicity, accelerated tumour growth, delayed treatment options and unnecessary costs [5, 6]. If reliable efficacy predictors are available before or early in treatment to help physicians develop individualised treatment regimens, accurate treatment regimens may be considered for patients who may not be completely cured or have poor prognosis. Accurate prediction can not only provide decision basis for drug treatment and avoid incorrect medication but also provide guidance for patients insensitive to conventional treatment to change the drug type, adjust radiation dose and CCRT regimen or receive surgery immediately to avoid delayed treatment opportunity and waste of time and money [6, 7]. Therefore, the accurate prediction of the sensitivity and tolerance of tumour cells is the key to clinical treatment.

With the rapid development of magnetic resonance imaging (MRI) technology, repeated and noninvasive evaluation of tissue characteristics has become one of the main methods in the field of cervical cancer [8–10]. Conventional T1-weighted images (T1WIs) and T2WIs can provide fine morphological information on lesions. New technologies, such as diffusion-weighted imaging (DWI) and dynamic contrast enhanced-MRI (DCE-MRI), provide additional tissue metabolic information and thus have the potential to be used as early predictors. Traditional medical imaging mode based on morphological changes limits the image analysis to a range of visual judgement. However, the resolution of human eyes is limited; thus, detecting all fine and tiny features in images is difficult [11–13]. The predictive capability of traditional imaging techniques for tumour response to treatment is still extremely limited.

The organic integration of big data technology and medical image-assisted diagnosis has promoted the emergence of a new imaging method, that is, radiomics [14–16]. By extracting massive features from images and capturing potential intra-tumour heterogeneity to predict treatment response, radiomics can effectively solve the problem of difficulty in quantitative evaluation of tumour heterogeneity and guide the formulation of personalised treatment plans [17, 18]. Single or combined imaging radiomics features can be used to guide accurate diagnosis, establish potential prognostic models and propose effective treatment strategies [19–21]. However, they only extract texture features and intensity histogram for analysis but do not establish a prediction model nor give full play to the powerful function of image radiomics. Random forest is an accurate and efficient method among many imaging radiomics methods, and it has been applied to the evaluation and efficacy prediction of a variety of tumours [22–24]. However, studies on the efficacy prediction of CCRT for cervical cancer are limited. This study aimed to predict the efficacy of CCRT for cervical cancer through MR image analysis before treatment with random forest algorithm.

## Methods and materials

### Clinical data

**Fig. 1:**
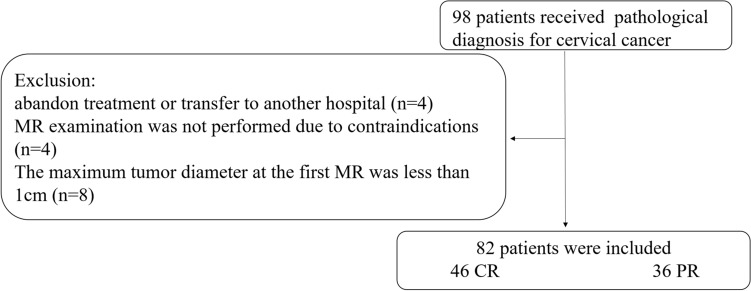
Recruitment pathway for patients in this study. Ninety-eight patients received pathological diagnosis for cervical cancer were included initially. Sixteen patients were excluded according to the exclusion criteria, and a total of 82 patients were eventually included in the study. *CR* complete response, *PR* partial response

This study is a retrospective case–control research. Data collection protocols were approved by the Ethics Committee of Qinhuangdao Municipal No.1 hospital, and the need for informed consent was waived. From March 2013 to May 2018, 82 cervical cancer patients aged 29–68 years with an average age of 52.6 years were recruited from Qinhuangdao Municipal No.1 Hospital. The clinical data of all patients, among which 74 were squamous cell carcinoma, and 8 were adenocarcinomas, were complete and pathologically confirmed. The clinical FIGO stage types were as follows: 40 IIb cases, 23 IIIa cases, 10 IIIb cases, 4 IVa cases and 5 IVb cases. The inclusion criteria were as follows: (1) The cervical cell pathological smear was diagnosed with cervical cancer, but patients with stage II B–IV cannot undergo surgery treatment. (2) Patients did not receive any other treatment before completing the CCRT programme, and co-occurrence of another malignant tumour was not observed during the study. (3) Pelvic MRI examination was completed within 2 weeks before and 3 months after treatment. The exclusion criteria were as follows: (1) patients abandoned the treatment or transferred to another hospital; (2) presence of contraindications due to MRI (such as cardiac pacemaker and neurostimulator, intrauterine device and claustrophobia and severe history of gadolinium allergy, etc.) and failure to undergo MR examination; (3) maximum tumour diameter of less than 1 cm and region of interest (ROI) less than 3 layers, thus preventing three-dimensional (3D) imaging. The data were divided into training and test sets by means of stratified sampling at a ratio of 4:1. The flow chart is as follows (Fig. 1).

### MRI Examination methods

All patients received routine pelvic MRI sequence scanning within 2 weeks before and 1 month after treatment. Siemens Avanto 1.5 T MR scanner (Siemens, Munich, Germany) with body coil was used for scanning. The patient lay headfirst in supine position. The main sequences included the following: sagittal fat saturation T2WI, coronal fat saturation T2WI, horizontal fat saturation T2WI, horizontal echo-planar imaging-acquired DWI and horizontal 3D volumetric interpolated breath-hold examination. Table 1 provides the specific parameters. The orientation of the scan was determined based on the cervical shape and position (forward bend, backward bend and upright), and scanning was performed at an angle perpendicular to or parallel to the cervix to obtain the maximum layer of cervical cancer.

**Table 1:** MRI protocol for endometrial cancer

Sequences parameters	MRI sequences				
	Sagittal-T2WI	Coronal-T2WI	Axial-T2WI	Axial-DWI	Axial-3D-VIBE
Fat saturation	Yes	Yes	Yes	Yes	Yes
TR/TE (msec)	4340/92	4340/92	4340/92	75/2.38,4.79	4.44/2.16
Angle (°)	150	150	150	70	10
Slice thickness (mm)	4	4	4	4	3
FOV (mm^2^)	280	280	280	280	280
Voxel Size (mm^3^)	0.6 × 0.6 × 4.0	0.6 × 0.6 × 4.0	0.6 × 0.6 × 4.0	1.6 × 1.6 × 4.0	0.6 × 0.6 × 3.0
Interslice gap	10%	10%	10%	10%	0
Delay (s)					0, 25, 60, 180
Scan time (s)	145	145	145	130	17
b-Value (s/mm^2^)			0, 800		

*FOV* field of view

### Treatment plan and efficacy evaluation

CCRT: (1) 6MV-X ray was used in radiotherapy. For the pelvic 3D conformal intensity-modulated radiation therapy, the primary tumour areas, such as uterus, cervix and vagina, and the total iliac, internal and external iliac, obturator and anterior sacral lymph nodes were used as clinical target volume (CTV). The external CTV radiation of 10 mm was used as planned target volume. The dose was 180–200 cGy/time treatment for 5 times a week, and the cumulative dose (DT) was 3000 cGy/15–17 times. For intracaval afterloading treatment, the radiation source was 192Ir, the dose was 600 cGy/time, and the DT was 3600–4200 cGy/6–7 times. The depth of the applicator into the uterine cavity should be determined based on the specific condition of the patient, the radiotherapy dose should be determined based on the tumour size and the dose should be relatively high for large tumours. (2) For chemotherapy, on the basis of radiotherapy, cisplatin (40 mg/m^2^) was administered simultaneously once a week with intravenous infusion for a total of 6 weeks of chemotherapy.

Efficacy evaluation: Tumour was observed in each MR sequence after radiotherapy. The efficacy results of radiotherapy and chemotherapy for tumours were divided into two groups in accordance with the Response Evaluation Criteria In Solid Tumours (RECIST): (1) complete response (CR) group, the tumour completely disappeared; (2) partial response (PR) group, the tumour shrank but did not disappear completely. No tumour progression was observed in all cases in this study.

### Image segmentation and radiomics feature extraction

#### Segmentation

The original images in Digital Imaging and Communications in Medicine format of each scan sequence of all enrolled patients before chemoradiotherapy were derived from picture archiving and communication system. Before the quantitative imaging features were extracted, ITK-SNAP tool was used to manually segment the sagittal fat-suppression T2WIs layer by layer, and 3D volume of interest was generated after the outline was completed. Manual segmentation of all tumour areas was performed by a radiologist with 10 years of diagnostic experience. The ROI was drawn along the edge of the lesion, and image analysis was carried out in accordance with the following points: (1) careful observation of the size, shape and edge of the lesion, attempted fitting of the tumour boundary during sketching and sketching of the tumour area as completely as possible; (2) sketching only the area invaded by cervical tumour to assess whether uterine body and vagina are involved; (3) inclusion of lesions with liquefaction, necrosis and cystic degeneration in the ROI range as they were also part of the tumour appearance; (4) patients who showed no clear boundary and edge of cervical cancer lesions on conventional MRI and DWI should be sketched against each other’s scan sequences at the same level; (5) avoidance of mucus in the cervix; (6) sketching of the sagittal T2 image and referring to DWI and dynamic enhanced scan when the tumour boundary and invasion scope were unclear.

#### Extraction

**Fig. 2:**
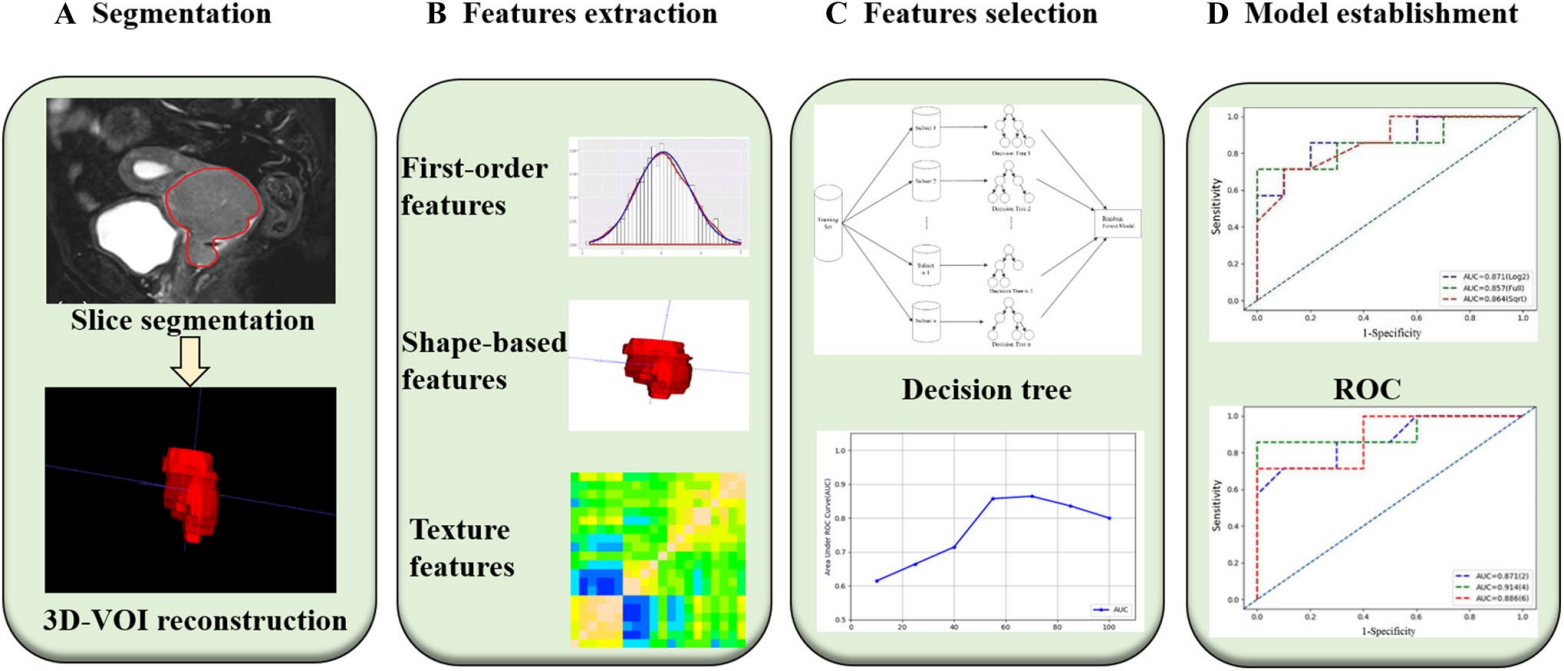
Radiomics workflow of model construction. **a** MR images segmentation. First, the tumour was segmented manually on the sagittal image, and then ITK-SNAP was used for 3D volume reconstruction. **b** Radiomic feature extraction. According to the segmentation image, a total of 106 radiomics parameters of 3 types were extracted from each set of images. **c** Radiomic Feature selection. After the preprocessing of Wavelet Filtering and Laplacian of Gaussian, the characteristic parameters were selected and classified by decision-making tree. **d** Model establishment. The diagnostic efficacy of the radiomics model was evaluated by ROC analysis

Imaging radiomics features were extracted using Pyradiomics (https://pyradiomics.readthedocs.io/en/latest/index.html), an open source tool based on the Python platform. First, wavelet filtering and Laplacian of Gaussian were used to preprocess the images. A total of 106 characteristics in 6 categories were extracted for the original image and the image preprocessed by wavelet transform and Gaussian Laplace operator, including the first-order features (*n* = 19), shape-based features (*n* = 26), grew up around grey level co-occurrence matrix (*n* = 24), grey level size zone matrix (*n* = 16), grey level run length matrix (*n* = 16); grew up around grey tone difference matrix (*n* = 5). The characteristics of first-order statistics were obtained by using common indicators, such as entropy, minimum grey value and variance of grey value, to calculate the grey value distribution of pixels in tumour areas in MRI images. Shape feature is the characteristic description of tumour area volume, area and maximum diameter in MRI images of cervical cancer. Grey co-occurrence matrix, grey level size zone matrix, grey level run length matrix; grew up around grey tone difference matrix were used to describe the texture features of MRI images of cervical cancer by studying the spatial characteristics of grey scales and their relations. Intra-group correlation coefficients of the omics parameters measured by two radiologists were calculated for characteristic stability analysis. Figure 2 shows the specific process of image segmentation and omics feature extraction.

### Construction of random forest model

**Fig. 3:**
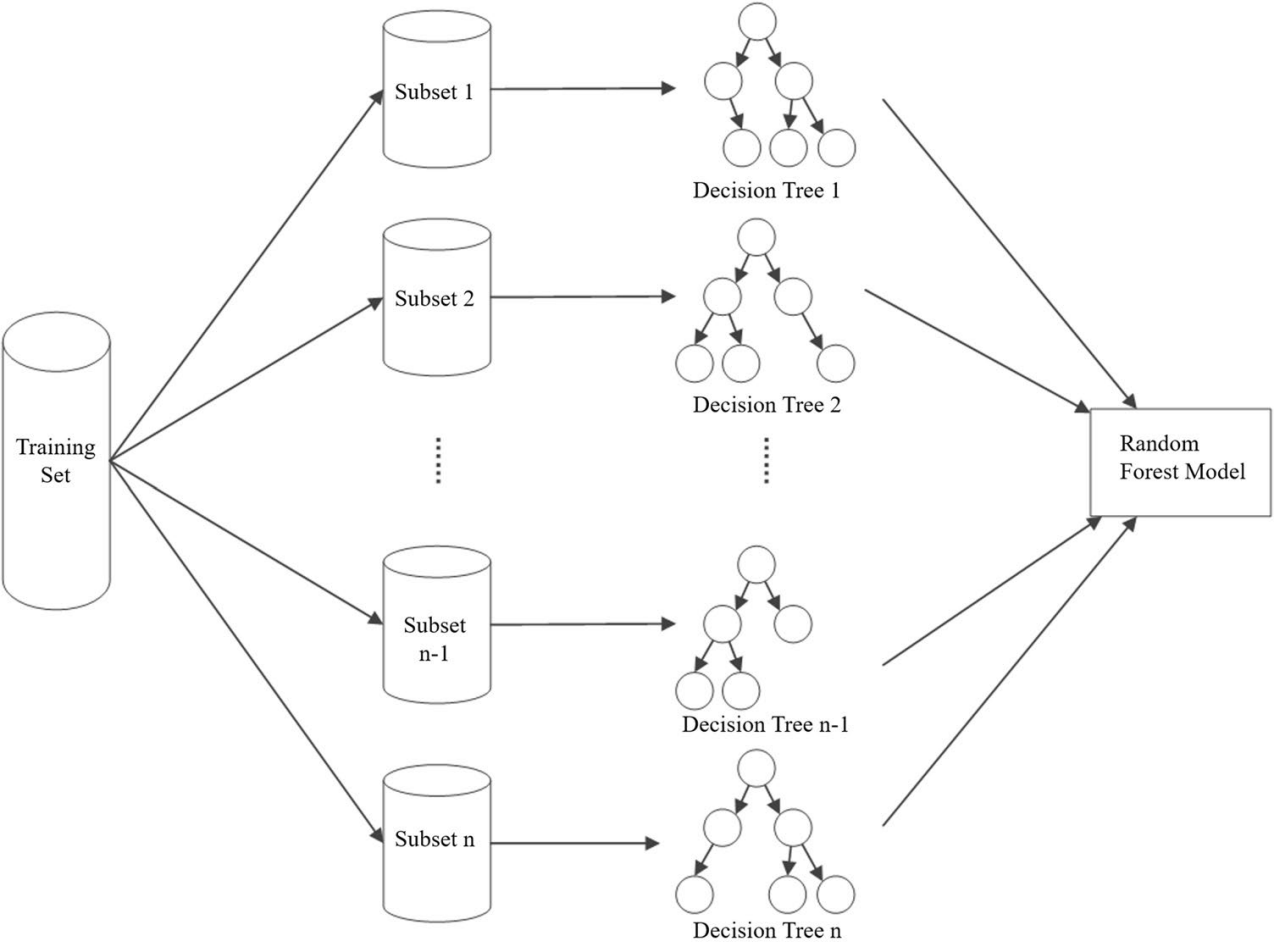
The development process diagram of random forest model. The training set is divided into N training subsets, and each subset generates a decision tree. A total of N decision trees are generated, and the n decision trees are assembled together. This process is the construction process of the random forest model

The random forest model was built and optimised on the open source toolkit scikit-learn (https://scikit-learn.org/stable/). After the image radiomics features are extracted, random samples are selected and all the radiomics features are put together to form the training sample set. Then, the training subset containing M samples is obtained through m times of put back sampling from the training sample set. In the construction process of the random forest, a total of N training subsets were collected, each containing M samples, and a decision tree was generated for each training subset. The N decision trees obtained from n training subsets constituted the random forest model. At the same time, in the process of constructing the random forest, this study attempted to compare the number of decision trees used in constructing the random forest model by using the optimal partitioning attribute selection method in the generation process of the decision trees. Figure 3 shows the construction process of the random forest model.

### Statistical methods

SPSS 17.0 statistical software was used for statistical analysis. *T*-test, one-way analysis of variance and Student–Newman–Keuls were used for comparison. *p* < 0.05 was considered statistically significant.

## Results

**Fig. 4:**
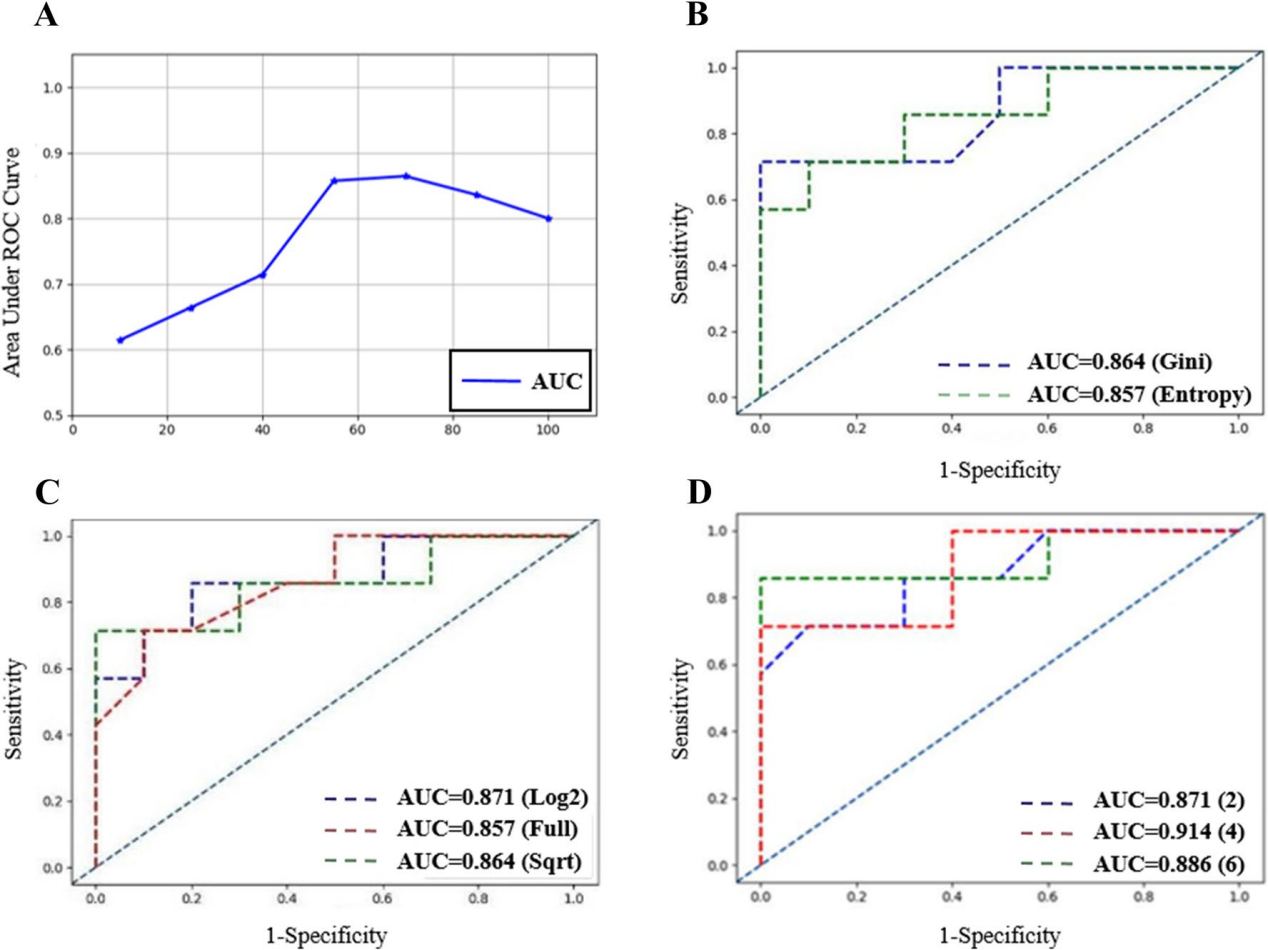
**a** The relationship between the number of decision trees in random forest and the performance of the model. When the number of decision trees was set to 10, 25, 40, 55, 70, 85 and 100, respectively, the performance of random forest model shows a trend of rising first and then declining, and its inflection point appeared at 70. **b** Area under curve (AUC) at different final partition index. The Gini index demonstrated a better effect compared with Information gain index. For Gini index model, the AUC is 0.864. **c** AUC at different number of sub-samples (Nsub) randomly sampled for each decision tree in training. The best performance was acquired when Nsub was equal to the *log*_*2*_*N* which AUC is 0.871. **d** Area under curve (AUC) at different minimum number of samples (Nmin) partitioned by each node. The best performance was acquired when Nmin was equal to 6 which AUC is 0.914

1. In this study, baseline characteristics of all patients were grouped based on CR and PR, and no statistically significant differences were observed in age, pathological classification and grade of cervical cancer between the two groups (*p* values were 0.689, 0.714 and 0.984, respectively; Table 2).
2. After selection and classification, a variety of first-order parameters, shape-based features and texture parameters were different between CR and PR groups. Table 3 shows the specific parameters.
3. When the number of decision trees was set to 10, 25, 40, 55, 70, 85 and 100, the performance of the random forest model showed an initially rising and then declining trend, and its inflection point appeared at 70 (Fig. 4a).
4. The area under the receiver operating curve (AUC) of Gini index and information gain ratio were 0.864 and 0.857, respectively (Fig. 4b).
5. Four random forest models of the construction to generation process of decision trees can use the numbers of one of the largest characteristics, which is set to N with *log*_*2*_*N* and $\sqrt N$. The AUC values were 0.871, 0.857 and 0.864 in the process of decision-tree structure; they are the largest characteristic quantities that can be used to set *log*_*2*_*N* cases to achieve a better prediction effect (Fig. 4c).
6. For the comparison of the minimum sample sizes set to 2, 4 and 6, the AUC values were 0.871, 0.914 and 0.876, respectively (Fig. 4d).

**Table 2:** Baseline characteristics of cervical cancer patients

Characteristics	Total (*n* = 82)	CR (*n* = 46)	PR (*n* = 36)	*p*-value
Age (years)				0.689
Mean ± SD	52.6 ± 20.1	53.8 ± 23.2	51.7 ± 23.6	
Range	29–68	29–66	41–68	
Pathological type	82			0.714
Adenocarcinoma	74	42	32	
Squamous carcinoma	8	4	4	
FIGO				0.984
IIb	40	25	15	
IIIa	23	13	10	
IIIb	10	6	4	
IVa	4	2	2	
IVb	5	3	2

**Table 3:** Radiomics signatures and statistical results between complete response group and partial response groups

	Complete respones (*n* = 46)	Partial respones (*n* = 36)	*t* value	*p* value
original_shape_MajorAxisLength	49.627 ± 14.242	56.216 ± 13.290	− 2.140	0.045
original_shape_Maximum2DDiameterRow	54.013 ± 16.089	61.185 ± 15.392	− 2.041	0.033
original_shape_Maximum2DDiameterSlice	53.422 ± 15.812	62.449 ± 15.365	− 2.597	0.0011
original_shape_Maximum3DDiameter	60.579 ± 18.341	69.023 ± 16.565	− 2.158	0.034
original_shape_MinorAxisLength	39.088 ± 11.188	44.195 ± 11.527	− 2.024	0.048
original_shape_SurfaceVolumeRatio	0.219 ± 0.0642	0.186 ± 0.045	2.591	0.011
original_glrlm_GrayLevelNonUniformity	49.961 ± 49.741	75.105 ± 56.788	− 2.134	0.036
original_glrlm_LongRunEmphasis	1.131 ± 0.067	1.170 ± 0.082	− 2.351	0.021
original_glrlm_RunLengthNonUniformityNormalized	0.925 ± 0.031	0.907 ± 0.036	2.422	0.018
original_glrlm_RunPercentage	0.960 ± 0.017	0.950 ± 0.021	2.407	0.018
original_glrlm_RunVariance	0.045 ± 0.024	0.059 ± 0.030	− 2.354	0.021
original_glrlm_ShortRunEmphasis	0.970 ± 0.013	0.962 ± 0.015	2.387	0.019
original_glszm_ZonePercentage	0.614 ± 0.117	0.548 ± 0.122	2.455	0.016
original_gldm_DependenceNonUniformityNormalized	0.329 ± 0.082	0.281 ± 0.079	2.663	0.009
original_gldm_DependenceVariance	1.550 ± 0.999	2.161 ± 1.306	− 2.399	0.019
original_gldm_GrayLevelNonUniformity	53.853 ± 53.904	81.930 ± 64.682	− 2.143	0.035
original_gldm_LargeDependenceEmphasis	5.879 ± 3.256	7.753 ± 4.135	− 2.296	0.024
original_gldm_SmallDependenceEmphasis	0.550 ± 0.105	0.489 ± 0.108	2.567	0.012
original_ngtdm_Busyness	0.051 ± 0.046	0.085 ± 0.074	− 2.514	0.014
original_ngtdm_Coarseness	0.008 ± 0.006	0.005 ± 0.004	2.220	0.014
original_firstorder_Mean	176.352 ± 74.562	135.824 ± 93.958	2.118	0.038
original_firstorder_Median	177.500 ± 79.431	130.046 ± 91.160	2.516	0.014
original_firstorder_Minimum	− 39.735 ± 0.32.416	− 54.640 ± 32.348	2.3068	0.042
original_firstorder_RootMeanSquared	481.970 ± 76.421	440.673 ± 96.611	2.101	0.039
original_glcm_ClusterShade	7,054.624 ± 14,680.498	19,656.274 ± 35,256.798	− 2012	0.050
original_glcm_DifferenceAverage	10.035 ± 3.281	8.409 ± 3.357	2.204	0.030
original_glcm_Id	0.216 ± 0.053	0.246 ± 0.062	− 2.303	0.024
original_glcm_Idm	0.135 ± 0.049	0.162 ± 0.060	− 2.257	0.027
original_glcm_Imc1	− 0.254 ± 0.11956	− 0.179 ± 0.078	− 3.458	0.001
original_glcm_Imc2	0.929 ± 0.075	0.888 ± 0.070	2.533	0.013
original_glcm_InverseVariance	0.139 ± 0.050	0.167 ± 0.059	− 2.263	0.026
original_glcm_MCC	0.722 ± 0.088	0.656 ± 0.091	3.336	0.001

## Discussion

Considerable radiomics analyses have been performed on DWI and DCE-MRI but rarely on T2WI [25–28]. We believe that the sagittal T2WI can better display the morphological structure and signal of the cervical canal and provide a good diagnostic condition for the display of lesions, lesion scope and evaluation of the invasion of surrounding tissues [29]. Cervical cancer lesions on T2WI appear as masses with moderate signal, and the truncation of low signal in fibrous matrix is one of the diagnostic criteria for lesions [30]. Fat people have considerable amount of fatty tissue in their pelvic cavity. Adipose tissue shows high signal on nonsaturation T2WIs, thus interfering with image observation. Fat suppression solves this problem by providing clear contrast, highlighting the diseased tissue to be observed and improving image quality. After the horizontal axis scanning of fat suppressed T2WI (FS-T2WI), the adipose tissue signal in the scanning range was reduced to a low signal, whereas the water-rich tissue signal remained unchanged to enhance the image contrast and the detection rate of lesions and reduce the interference of fat signal to the diagnosis. In addition, compared with DWI and DCE-MRI, T2 sequence has advantages in contrast and definition and can delineate the lesion area more accurately [31]. Therefore, this study attempted to analyse FS-T2WI, which has not been reported thus far.

Patankar et al. observed that the two-year overall survival rate of patients with CR (85.7%) to cervical cancer in the first 3 months after treatment was significantly higher than that of patients with PR (14.3%) [32]. Therefore, the treatment of cervical cancer currently pursues the cure rate rather than the effective rate. Whether the tumour can be completely alleviated within the first 3 months after treatment is the key to the prognosis of patients. In this study, the efficacy evaluation did not completely use traditional RECIST classification but only divided the patients into the CR and PR groups. In addition to the above reasons, no cases in our data showed lesion changes that were not evident or increased after treatment.

Radiomics is a developing field of research that aims to extract complex information from traditional medical images, including features that are not easily seen or quantified, to form a high-dimensional feature space that can be developed [33, 34]. Single or combined imaging omics features can guide accurate diagnosis, establish potential prognostic models and propose effective treatment strategies. The iterative random forest algorithm is a model of integrated learning that consists of a number of decision-tree classification machines [35]. Each tree is built up with independent sets that behave independently of each other. In the random forest model, parameter adjustment is less needed. Thus, overfitting is avoided, and the prediction accuracy is improved without significantly increasing the computation. The algorithm of random forest randomly extracts a certain number of samples from the features of the original training set for training. These samples are all randomly extracted with put back, new training model sets are produced and sub-data sets are constructed for construction. Then, the features to be classified are inputted into the model, and the final classification type of a feature is obtained by following the principle of the majority (i.e. the score obtained by the number of votes cast). All features are randomly selected, and the optimal features are obtained from the randomly selected features. In this way, the random forest is not prone to overfitting and has a good anti-noise capability. In addition, random forest can process high-dimensional data without feature selection, and the interaction between features can be detected during training. In this study, random sample selection and random attribute selection not only ensured that the performance of a single decision-tree model was not degraded, but it also reduced the correlation between different decision-tree models. Through combined strategies, such as average and voting methods, the stochastic forest model not only inherits the advantages of the decision-tree model but also greatly improves its prediction capability [36].

Random forest is a method used to achieve desirable generalisation performance by integrating a large number of decision trees [37]. The number of decision trees in a random forest influences the model performance [38]. In this research, the parameters, such as the optimal partition attribute selection criteria and the maximum characteristic number, were kept unchanged and the performances of therapy efficacy prediction algorithms were compared under different number of decision trees to study the influence of the number of decision trees. In the comparison of the effect of different numbers of decision trees on the performance of the chemoradiotherapy efficacy prediction algorithm, this paper compared the performances of the efficacy prediction algorithm when the numbers of decision trees were set to 10, 25, 40, 55, 70, 85 and 100. With the increase in the number of decision trees, the performance of the prediction algorithm for the efficacy of chemoradiotherapy increased. However, when the number of decision trees reached a certain level, the performance of radiotherapy efficacy prediction algorithm decreased. The reason is as follows: with the increase in the number of decision trees, the complexity of the random forest model gradually increased, which resulted in the strong fitting capability of the model. However, when the complexity of the model increased to a certain extent, the generalisation performance of the model degraded and overfitting occurred. In the following experiments, the number of decision trees was set to 70.

In the generation process of decision trees, different optimal partition attribute selection criteria have varying emphases [39]. In this study, the selection criteria of two optimal partitioning attributes, namely, information gain ratio and Gini index, were compared. The Gini index focuses on selecting attributes that make the partitioned data set purer, whereas the information gain ratio focuses on selecting attributes that provide more information [40]. In this study, the chemoradiotherapy efficacy prediction model with Gini index as the optimal partition attribute selection standard had slightly better performance. Thus, in the following experiments, Gini index was used as the optimal partition attribute selection standard.

The decision tree can be balanced between model complexity and generalisation performance by selecting appropriate pruning parameters. In this study, the performance of the chemoradiotherapy efficacy prediction algorithm was compared based on two parameters related to pruning: the maximum number of features and the minimum number of samples dividing nodes. The maximum number of features that can be used in the process of decision-tree construction affects the complexity of decision tree and the correlation between different decision-tree models in the random forest model. We supposed a total of N features available for the construction of the decision tree. In this study, the maximum number of features that can be used in the process of decision-tree generation was set as N, with *log*_*2*_*N* and $\sqrt N$. The comparison study showed that the prediction effect was better when the maximum number of features can be set as *log*_*2*_*N* in the process of decision-tree construction. In the process of decision-tree generation, the decision tree can be simplified by limiting the minimum number of samples in nodes. In this study, the minimum sample size was set to 2, 4 and 6 for comparison. The comparison experiment showed that with the increase in the minimum number of samples in the decision-tree node, the performance of the radiotherapy efficacy prediction algorithm based on random forest first increased and then decreased. The reason for this phenomenon is that with the increase in the minimum sample number in the decision-tree node, the complexity of the decision-tree model decreases and a moderate minimum sample number can balance the training and generalisation errors. After optimisation, when the number of decision trees was set to 70, and the selection criterion of the optimal partition index was set to Gini, the AUC value can reach 0.914.

### Limitations

(1) Small sample size. (2) All patients used the same scan sequence and parameters, and all data came from a single study centre to ensure the reproducibility and stability of the radiomics label. In future studies, additional samples will be added by including the patients who were excluded in the current study.

## Data Availability

Not applicable.
